# TRPV1 Hyperfunction Involved in Uremic Toxin Indoxyl Sulfate-Mediated Renal Tubular Damage

**DOI:** 10.3390/ijms21176212

**Published:** 2020-08-27

**Authors:** Chien-Lin Lu, Chun-Hou Liao, Kuo-Cheng Lu, Ming-Chieh Ma

**Affiliations:** 1 Division of Nephrology, Department of Medicine, Fu Jen Catholic University Hospital, New Taipei City 24205, Taiwan; janlin0123@gmail.com; 2 School of Medicine, Fu Jen Catholic University, New Taipei City 24205, Taiwan; liaoch22@gmail.com; 3 Divisions of Urology, Department of Surgery, Cardinal Tien Hospital, New Taipei City 23148, Taiwan; 4 Division of Nephrology, Department of Medicine, Taipei Tzu Chi Hospital, Buddhist Tzu Chi Medical Foundation, New Taipei City 23142, Taiwan; kuochenglu@gmail.com

**Keywords:** transient receptor potential vanilloid 1, indoxyl sulfate, aryl hydrocarbon receptor, arachidonate 12-lipoxygenase, 12(*S*)-hydroxyeicosatetraenoic acid, renal tubular damage

## Abstract

Indoxyl sulfate (IS) is accumulated during severe renal insufficiency and known for its nephrotoxic properties. Transient receptor potential vanilloid 1 (TRPV1) is present in the kidney and acts as a renal sensor. However, the mechanism underlying IS-mediated renal tubular damage in view of TRPV1 is lacking. Here, we demonstrated that TRPV1 was expressed in tubular cells of Lilly Laboratories cell-porcine kidney 1 (LLC-PK_1_) and Madin-Darby canine kidney cells (MDCK). IS treatment in both cells exhibited tubular damage with increased LDH release and reduced cell viability in dose- and time-dependent manners. MDCK, however, was more vulnerable to IS. We, therefore, investigated MDCK cells to explore a more detailed mechanism. Interestingly, IS-induced tubular damage was markedly attenuated in the presence of selective TRPV1 blockers. IS showed no effect on TRPV1 expression but significantly increased arachidonate 12-lipoxygenase (ALOX12) protein, mRNA expression, and 12(*S*)-hydroxyeicosatetraenoic acid (12(*S*)-HETE) amounts in a dose-dependent manner, indicating that the ALOX12/12(*S*)-HETE pathway induced TRPV1 hyperfunction in IS-mediated tubulotoxicity. Blockade of ALOX12 by cinnamyl-3,4-dihydroxy-α-cyanocinnamate or baicalein attenuated the effects of IS. Since aryl hydrocarbon receptor (AhR) activation after IS binding is crucial in mediating cell death, here, we found that the AhR blockade not only ameliorated tubular damage but also attenuated ALOX12 expression and 12(*S*)-HETE production caused by IS. The uremic toxic adsorbent AST-120, however, showed little effect on ALOX12 and 12(*S*)-HETE, as well as IS-induced cell damage. These results clearly indicated that IS activated AhR and then upregulated ALOX12, and this induced endovanilloid 12(*S*)-HETE synthesis and contributed to TRPV1 hyperfunction in IS-treated tubular cells. Further study on TRPV1 may attenuate kidney susceptibility to the functional loss of end-stage kidney disease via IS.

## 1. Introduction

As a highly perfused organ, the kidney plays an important role in the maintenance of body homeostasis via tubular function to reabsorb water, nutrients, and electrolytes back to circulation and to excrete metabolic waste products. Renal tubules, however, are susceptible to ischemia injury, endogenous or exogenous toxins, and proteinuria. Transient receptor potential vanilloid type-1 (TRPV1) was cloned firstly in 1997 from the rat dorsal root ganglia (DRG) [1]. TRPV1 is found mainly in the nociceptive neuron of the peripheral nervous system and acts as a sensor in detecting noxious stimuli. It is a non-selective cation channel that is exclusively localized in mammalian tissue, predominantly in the nervous system, which allows for the influx of calcium and sodium into cells to depolarize neurons, leading to the perception of pain. In addition to the peripheral and central terminals of the sensory neuron, TRPV1 cDNA has also been cloned in nonneuronal cells, including in the kidney [2,3]. We previously showed that TRPV1 activation in the renal pelvis stimulated afferent renal nerve activity via substance P release, which then inhibited efferent renal sympathetic nerve activity and, finally, enhanced diuresis and natriuresis through a renorenal reflex mechanism [4]. Activation of TRPV1 in the isolated kidney decreases renal perfusion pressure and increases the glomerular filtration rate (GFR) and diuretic response [5]. These results indicate that TRPV1 plays an important role in maintaining renal hemodynamics and excretory function for keeping body fluid homeostasis. TRPV1 is also found to be expressed in renal tubules [4]; however, the effect of tubular TRPV1 distribution is still unclear.

Indoxyl sulfate (IS) is accumulated in serum due to inadequate renal clearance in chronic kidney disease (CKD), is associated with cardiovascular morbidity [6] and reduced bone density [7], and acts as a causative factor in the pathogenesis of CKD progression. Until now, the mechanisms by which IS induces nephrotoxicity have been widely studied. IS-induced renal epithelial cell death and epithelial-to-mesenchymal cell transition (EMT) change have been proposed. In renal proximal tubular human kidney-2 (HK-2) cells, IS increases proapoptotic protein expression, which induces tubular cell apoptosis and reduces cell viability [8]. In addition, IS also upregulates transforming growth factor-β and fibronectin expression and induces pro-inflammatory cytokines, such as interleukin-6 and tumor necrosis factor-α, which lead to renal interstitial fibrosis accompanied by tubule cell apoptosis [9]. Furthermore, IS promotes EMT change in renal epithelial cells, and extracellular matrix deposition drives renal fibrosis development and aggravates kidney function decline [10]. 

The toxic effect of IS is mediated by binding to the aryl hydrocarbon receptor (AhR). AhR is a transcription factor that regulates gene expression after binding to its response elements throughout the genome. AhR has been reported to modulate genes involving eicosanoid metabolism [11]. Arachidonate lipoxygenase (ALOX) metabolizes arachidonic acid (AA) and produces eicosanoids to provoke diverse responses, including inflammation in kidney diseases. Arachidonate 12-lipoxygenase (ALOX12) produces 12-hydroperoxyeicosatetraenoic acid and, subsequently, rapidly reduces to 12-hydroxyeicosatetraenoic acid (12(*S*)-HETE) [12]. 12(*S*)-HETE demonstrates a diverse renal effect; it mediates vasoconstriction response to angiotensin II and decreases renal blood flow (RBF) and GFR, with the intrarenal arterial treatment of 12(*S*)-HETE [13,14]. 12(*S*)-HETE also causes mesangial cell constriction and compromise changes in RBF. Moreover, 12(*S*)-HETE inhibits proximal tubular Na^+^, K^+^-ATPase activity, and sodium reabsorption [15]. 12(*S*)-HETE also acts as an endogenous ligand for TRPV1 [16] and is known to inhibit renin release via the effect of afferent renal nerve activity [17]. Although the diverse effect of 12(*S*)-HETE in renal dysregulation has been discovered, its impact on renal tubular cells has not yet been studied.

Studies have shown tubular dysfunction in renal insufficiency, and this has been associated with pathophysiological deterioration in several renal disorders, such as salt-induced kidney damage [18,19], ischemia/perfusion injury [5,20,21], and fibrosis [22]. Thus, we hypothesized that TRPV1 is involved in IS-induced cytotoxicity in renal tubular cells in CKD. However, the literature review concerning TRPV1-mediated renal tubular cells’ injury is lacking. Moreover, the mechanism by which IS-induced renal tubular cell cytotoxicity occurs has not yet been fully elucidated. Therefore, this study aimed to investigate the mechanism of IS-induced renal tubular damage in regard to TRPV1. 

## 2. Results

### 2.1. Indoxyl Sulfate Induces Renal Tubule Epithelial Cell Cytotoxicity

**Figure 1:**
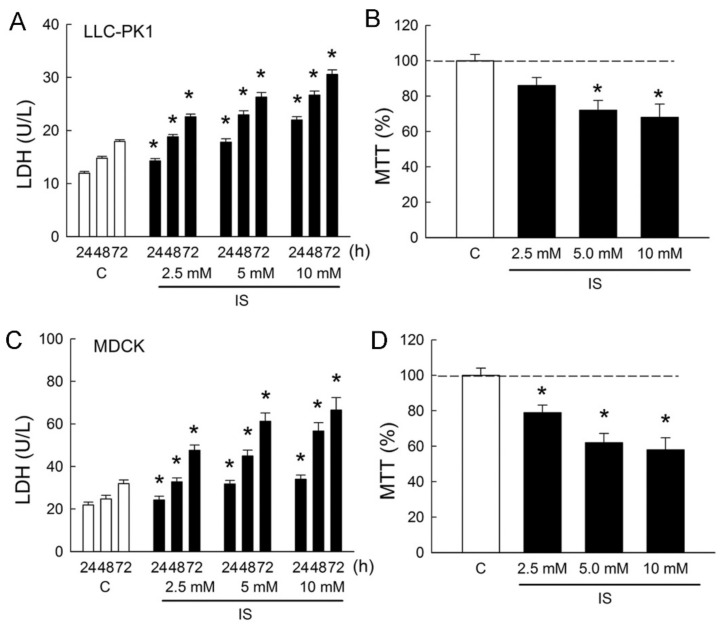
Indoxyl sulfate induces cytotoxicity in tubular cells. (**A**,**C**) Lilly Laboratories cell-porcine kidney 1 (LLC-PK_1_) and Madin-Darby canine kidney cells (MDCK) were treated with phosphate-buffered saline (PBS) or indoxyl sulfate (IS, 2.5, 5.0, and 10.0 mM) for 24, 48, and 72 h. Lactate dehydrogenase (LDH) release was measured as a marker of cell injury; (**B**,**D**) Both cell lines were cultured for 72 h in the presence of PBS or IS, and the cell viability was monitored in an MTT assay. *n* = 6 of experiments performed at each time point and dose. * *p* < 0.05 versus the control group (**C**) at the same time point.

In order to determine whether IS induces cytotoxicity in renal tubular cells, Lilly Laboratories cell-porcine kidney 1 (LLC-PK_1_) and Madin-Darby canine kidney cells (MDCK) were used as representatives for the renal proximal tubule and distal tubule segment of the nephron, respectively. Treatment of cells with IS led to dose- and time-dependent increases in lactate dehydrogenase (LDH) release from LLC-PK_1_ and MDCK cells (Figure 1A,C). In the LLC-PK1 cells, LDH increased to 189 ± 7%, 220 ± 16%, and 255 ± 24% when compared to those in the control group after 72 h of IS treatment (all *p* < 0.05). In the MDCK cells, LDH increased to 217 ± 29%, 279 ± 32%, and 313 ± 42% when compared to those in the control group after 72 h of IS treatment (all *p* < 0.05). In addition, IS also exhibited a reduction in cell survival in both LLC-PK_1_ cells and MDCK cells in a dose-dependent manner after 72 h of treatment. In the LLC-PK_1_ cells, significant reductions in cell viability were observed at 5 and 10 mM of IS treatment (Figure 1B). In the MDCK cells, cell viability was significantly reduced at all doses after IS treatment (Figure 1D). These results clearly indicated that IS possessed a potent cytotoxicity effect on both proximal and distal tubule cells and that MDCK was more vulnerable to IS than LLP-CK_1_. We, therefore, chose MDCK cells to investigate the cellular mechanism related to IS-induced cytotoxicity in the following experiments.

### 2.2. Transient Receptor Potential Vanilloid 1 (TPRV1) Is Expressed in Tubular Cell Lines

**Figure 2:**
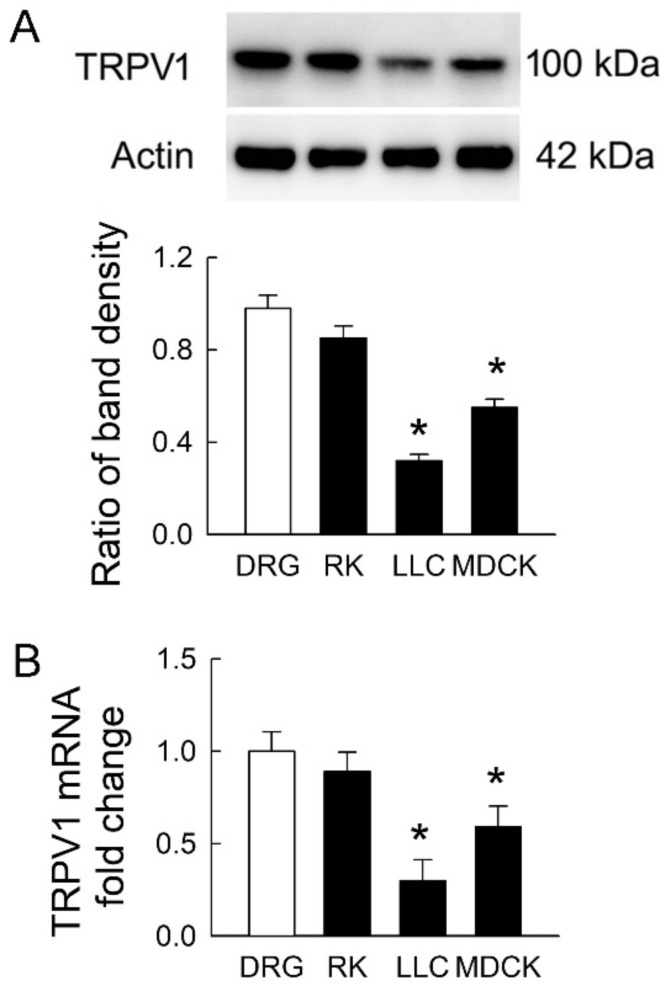
Transient receptor potential vanilloid type-1 (TRPV1) expression in tubular cell lines. (**A**) Representative immunoblots show TRPV1 expression in rat dorsal root ganglia (DRG) tissues, rat kidney (RK), LLC-PK_1_ cells (LLC), and MDCK cells using 20 μg of the total protein in each lane. The lower bar graph shows densitometrically quantified results by the ratio of TRPV1 to β-actin; (**B**) The expression of TRPV1 mRNA in rat tissues and tubular cells was examined by real-time quantitative RT-PCR. *n* = 4 of experiments performed in each group. * *p* < 0.05 versus DRG.

TRPV1 is widely distributed in the rat kidney [4]. It is still unclear whether TRPV1 is present in LLC-PK_1_ and MDCK cells. Using rat DRG and kidney tissues as positive controls, we found that TRPV1 protein was expressed in both renal tubular cells (the representative blots in Figure 2A). However, the extent of TRPV1 protein expression in both cell lines was significantly lower than that in DRG. The levels of TRPV1 were 32 ± 6% and 56 ± 9% in LLC-PK_1_ and MDCK cells compared to those in DRG, respectively (the lower bar graph). The TRPV1 mRNA showed similar expression to that in protein levels (Figure 2B). These results demonstrated that transient receptor potential vanilloid 1 (TPRV1) in MDCK cells was more abundant than that in LLC-PK_1_ cells and indicated that MDCK was more vulnerable to IS if TRPV1 was the downstream pathway in this cytotoxicity.

### 2.3. Blockade of TPRV1 Attenuates Indoxyl Sulfate (IS)-Induced Cytotoxicity

**Figure 3:**
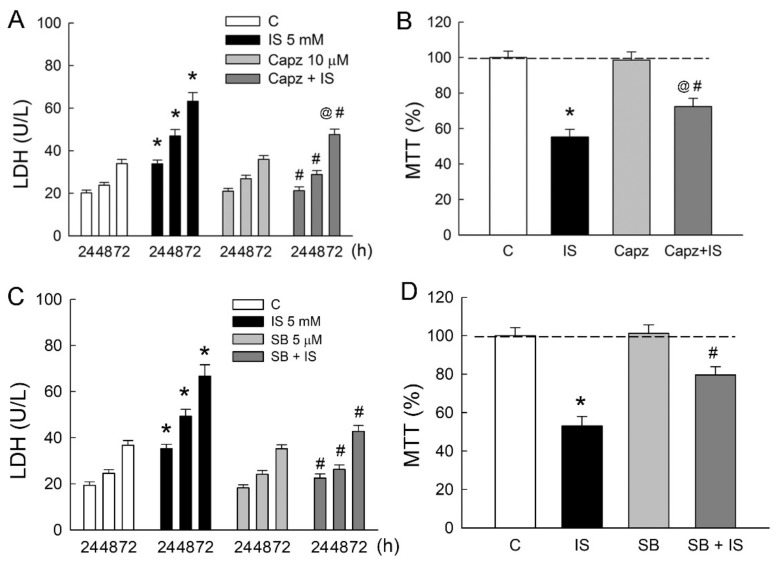
Blockade of TRPV1 attenuates indoxyl sulfate-induced tubular cell damage. MDCK cells were treated with a selective TRPV1 blockers capsazepine (Capz, 10 μM), SB-366791 (SB, 5 μM), or indoxyl sulfate (IS, 5 mM) alone or in combination for 24, 48, and 72 h. (**A**,**C**) Lactate dehydrogenase (LDH) was determined as a marker of cell injury; (**B**,**D**) Cells were cultured for 72 h in the presence of the indicated agents, and the viability was measured in an MTT assay. *n* = 6 of experiments performed in each time point of the group. * *p* < 0.05 versus the control group (C), ^@^ *p* < 0.05, Capz (or SB) + IS versus Capz (or SB) group, ^#^ *p* < 0.05, Capz (or SB) + IS versus IS group.

To determine whether TRPV1 is involved in IS-induced tubular damage in MDCK cells, cells were treated either with IS alone or in combination with TRPV1 blocker capsazepine. Similar to the above results, 5 mM IS significantly led to an increase in LDH release in a time-dependent manner (Figure 3A). In co-treatment with capsazepine (Capz), LDH released from IS-treated cells was markedly diminished as compared to those in the IS group. This was also associated with an increase in cell viability in the Capz + IS group when compared to that in the IS group (Figure 3B). The co-treatment of selective TRPV1 blocker SB-366791 with IS also significantly reduced LDH release and improved cell viability due to IS (Figure 3C,D). These results clearly indicated that TRPV1 played a significant role in the IS-induced cytotoxicity of renal tubular cells.

### 2.4. IS Enhances Arachidonate 12-Lipoxygenase (ALOX12) Expression and 12(S)-Hydroxyeicosatetraenoic Acid (HETE) Synthesis

**Figure 4:**
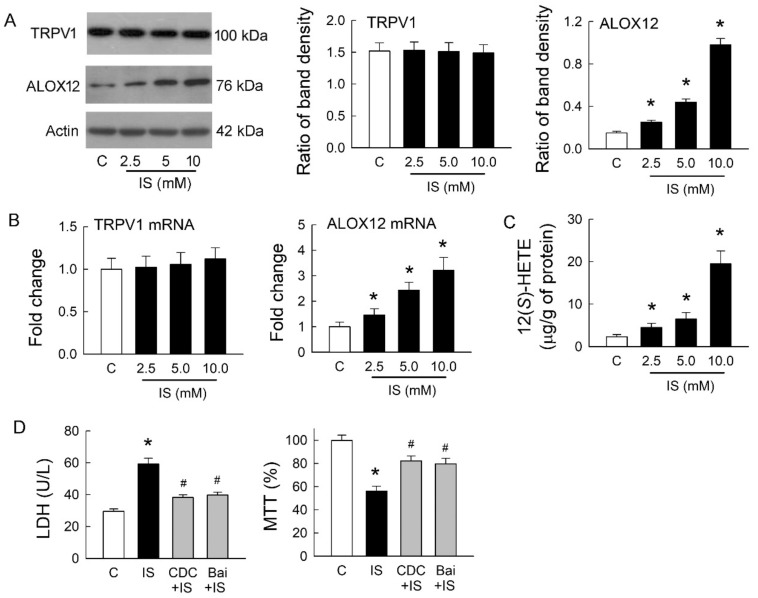
Effects of indoxyl sulfate on TRPV1 and arachidonate 12-lipoxygenase (ALOX12) expression. (**A**) Representative blots show the protein expression of TRPV1 and ALOX12 in MDCK cells with or without treatment of indoxyl sulfate (IS) using 20 μg of total protein in each lane. The right bar graphs show the ratio of TRPV1 or ALOX12 to β-actin (*n* = 6 per group). Note that IS treatment for 72 h did not affect TRPV1 expression but increased ALOX12 expression in a dose-dependent manner; (**B**) Changes in TRPV1 and ALOX12 mRNA were examined by real-time quantitative RT-PCR. Note that ALOX12 mRNA demonstrated a similar trend as its protein increased, caused by IS; (**C**) Changes in 12(*S*)-HETE levels in cells were determined by an enzyme-linked immunosorbent assay. Note that the level of 12(*S*)-HETE significantly increased after IS treatment in a dose-dependent manner; (**D**) Treatment of IS cells with selective ALOX12 inhibitors cinnamyl-3,4-dihydroxy-α-cyanocinnamate (CDC) or baicalein (Bai) reduced LDH release and enhanced cell viability by an MTT assay. *n* = 6 of experiments performed in each group. * *p* < 0.05 versus the control group (C), ^#^ *p* < 0.05 versus IS group.

We previously showed that endovanilloid 12(*S*)-HETE, a product of AA metabolized by ALOX12, could stimulate TRPV1 and mimic its effects in rat hearts [16]. Whether ALOX12 and 12(*S*)-HETE show a similar effect on TRPV1 after IS treatment was our interest. The representative blots showed protein expression of TRPV1 and ALOX12 in MDCK cells with or without treatment of IS. IS treatment at various doses for 72 h did not affect TRPV1 expression, but this increased ALOX12 expression in a dose-dependent manner (Figure 4A). Changes in TRPV1 and ALOX12 mRNA showed a similar result to those at both protein levels; IS showed no effect on TRPV1 but dose-dependently increased in ALOX12 mRNA expression (Figure 4B). The cellular content of 12(*S*)-HETE was also dose-dependently increased after IS treatment (Figure 4C). We then investigated whether inhibition of ALOX12 can reverse the cytotoxic effect mediated by IS (Figure 4D). Compared to the IS-treated cells, a selective ALOX12 blocker cinnamyl-3,4-dihydroxy-α-cyanocinnamate (CDC) significantly lowered LDH release and enhanced cell viability. Moreover, another ALOX12 blocker baicalein (Bai) to the IS-treated cells showed similar effects as those of the CDC by reducing LDH release and increasing cell viability. These results suggested that IS-induced tubular cell damage was dependent on the effect of ALOX12-mediated 12(*S*)-HETE generation, which is known as an endogenous ligand for TRPV1 activation. This also clearly indicated that TRPV1 overstimulation for channel hyperfunction was caused by 12(*S*)-HETE involved in IS-induced tubulotoxicity.

### 2.5. Roles of Uremic Toxin Adsorbent and Aryl Hydrocarbon Receptor (AhR) Inhibition in Tubular Cell Injury

**Figure 5:**
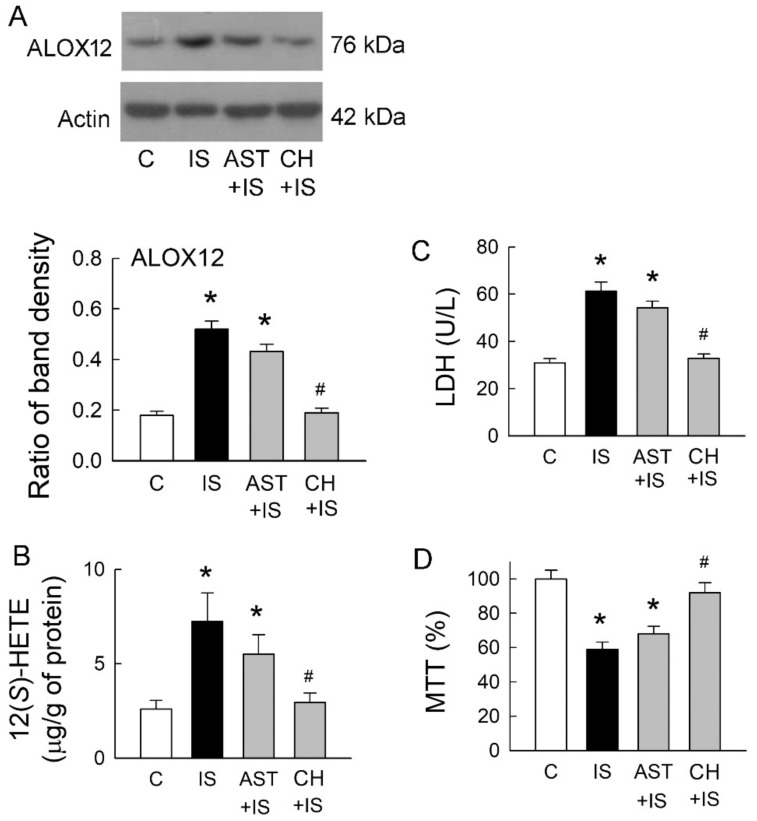
Effects of uremic toxin adsorbent (AST-120) and aryl hydrocarbon receptor (AhR) inhibitor on IS-treated cells. (**A**) Representative blots show the protein expression of ALOX12 in MDCK cells treated with IS (5 mM), uremic toxin adsorbent AST-120 (AST, 2 g/mL), AhR inhibitor CH-223191 (CH, 10 μM), and a combination of these drugs using 20 μg of the total protein in each lane. The lower bar graphs show the ratio of ALOX12 to β-actin (*n* = 6 per group); (**B**) Changes in 12(*S*)-HETE levels in cells were determined by an enzyme-linked immunosorbent assay; (**C**) Lactate dehydrogenase (LDH) was determined as a marker of cell injury; (**D**) Cells were cultured for 72 h in the presence of the indicated agents, and the viability was measured in an MTT assay. *n* = 6 of experiments performed in each group. * *p* < 0.05 versus the control group (C), ^#^ *p* < 0.05 versus IS group.

A previous study has demonstrated that IS is a potent endogenous agonist for AhR, and this effect can be neutralized by uremic toxin adsorbent AST-120 (AST) [7]. AhR activation regulates a diverse set of genes, including ALOX12 [11]. We, therefore, examined whether IS scavenge and AhR inhibition may affect ALOX12 expression, as well as 12(*S*)-HETE formation. Compared to the control group, ALOX12 upregulation in IS-treated cells was only slightly decreased after they were co-treated with AST-120 (Figure 5A). Interestingly, IS-mediated ALOX12 upregulation was significantly attenuated by a specific AhR inhibitor CH-223191 (CH). This was associated with a parallel change in the cellular contents of 12(*S*)-HETE (Figure 5B). We then examined whether changes in ALOX12 expression also affect IS-mediated cell damage. AST co-treated with IS had little effect on the reduction of LDH release, but the level of LDH release was similar to that in the control group when IS cells were co-treated with AhR inhibitor CH-223191 (Figure 5C). Compared with the IS group, IS co-treated with AST-120 had little effect on the cell viability (Figure 5D). AhR inhibition significantly rescued cells from IS-induced damage by increasing cell viability. These results clearly indicated that AhR inhibition ameliorated tubular cell damage caused by IS via the suppression of ALOX12 and 12(*S*)-HETE upregulations and potentiated the role of AhR in IS-mediated tubular cell injury.

## 3. Discussion

**Figure 6:**
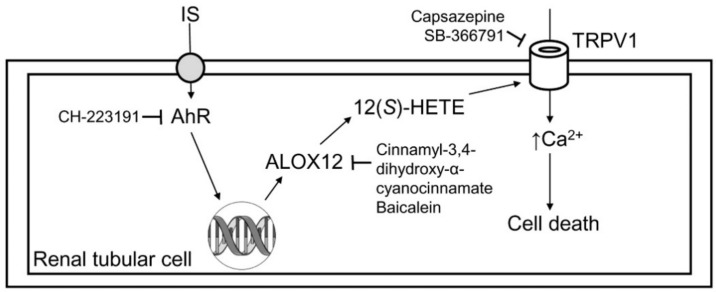
Schematic diagram showing how indoxyl sulfate induced cytotoxicity in tubular cells. After the uptake of indoxyl sulfate (IS), IS then induced tubular cell damage via AhR activation, and this effect could be abrogated by the AhR inhibitor CH-223191. AhR upregulated ALOX12 expression and cellular 12(*S*)-HETE synthesis. The blockade of ALOX12 also attenuated IS-mediated tubular damage. Excess formation of 12(*S*)-HETE overstimulated TRPV1 to induce cell damage, and this effect could be attenuated after TRPV1 inhibition by capsazepine and SB-366791.

As the schematic in Figure 6 demonstrates, IS directly damages renal tubular cells and reduces their viability. IS-induced tubular cell injury is associated with TRPV1 hyperfunction, which is proven by the use of TRPV1 blockers. The underlying mechanism for TRPV1 hyperfunction is related to AhR activation-mediated ALOX12 upregulation, and this increases the production of its metabolite 12(*S*)-HETE in tubular cells. The deleterious effect of IS on tubular cells can be abrogated by AhR inhibitor, which further supports the role of AhR in IS-mediated renal tubular damage.

Organic anion transporter (OAT) is located in renal tubules and functions as a solute exchanger to eliminate toxin (such as IS), drugs, and endogenous metabolites. OAT1 is mainly located in the S2 segment of the proximal renal tubule, while OAT3 is more broadly expressed in both S1 and S2 of the proximal tubule, the thick ascending limb of Henle, and the distal-convoluted tubule [23]. Tsuneo et al. reported that OAT3 was responsible for the transport of IS in the kidney and shared the same transport system with other uremic toxins [24]. The inhibitory effect of other uremic toxins to OAT3 transport impairs IS renal excretion and accelerates IS accumulation in CKD. In CKD, the tubular expression of OAT1 and OAT3 is greatly increased, and this is responsible for the secretion of accumulated uremic toxins by the rest of the nephrons [25]. Similarly, IS immunohistochemical staining is greatly increased in both proximal and distal renal tubules, which highlights the marked enhancement of the tubular uptake of IS in the remnant kidney [23]. Together with these findings, the elimination of accumulated IS in CKD is transported by enhanced OAT1 and OAT3 expression in remnant renal tubules, where IS might exert its nephrotoxic property on remnant tubular cells and fasten functional impairment [26]. Various types of OATs have been also found to be expressed in both LLC-PK_1_ and MDCK cells [27,28]. In this study, we found that IS exhibited nephrotoxicity over both LLC-PK_1_ and MDCK cells, which is representative of renal proximal and distal tubules, respectively, and more severe in MDCK cells. These findings are compatible with the in vivo distribution of OATs in renal tubules and the subsequent IS uptake into the cytoplasm by tubular cells.

TRPV1, also known as capsaicin receptor, is a nonselective ligand-gated ion channel with six transmembrane domains and a short, pore loop [29]. 12(*S*)-HETE is an endogenous ligand for TRPV1 that is capable of activating TPRV1 [30]. Using three-dimensional structure analysis, the carboxylic acid and hydroperoxide moiety in 12(*S*)-HETE share structural similarity to phenolic hydroxide and amide moiety in capsaicin, and, therefore, 12(*S*)-HETE has a high affinity to the capsaicin binding site of TRPV1 receptor [30]. After binding activation by 12(*S*)-HETE and capsaicin, TRPV1 can be equally permeable to calcium and sodium that evoke single-channel current, depolarizing the cell. Upon cellular injury, inflammatory mediators, such as prostaglandin or bradykinin, can activate protein kinase (PK)A or PKC-dependent pathways to potentiate capsaicin-mediated TRPV1 phosphorylation that lowers the temperature threshold of TPRV1 activation and enhances capsaicin-evoked response [31]. Furthermore, TRPV1 can be activated by ALOX12 products of AA; for example, TRPV1 in DRG neurons can be promoted by both 12(*S*)-HPETE and its reduced product 12(*S*)-HETE directly [30]. In mesencephalic dopaminergic neurons, 12(*S*)-HETE exhibits neuron cell death by promoting the TRPV1 channel opening and increases intracellular calcium concentration, leading to mitochondrial damage and cell death [32]. The influx of sodium and calcium via TRPV1 channels into pain-sensing cells causes cell swelling and impairs plasma membrane integrity, which leads to cell death [33]. 

The association between TRPV1 activation and epithelial cell damage in several tissues has been reported. Reilly et al. reported that TPRV1 activation by calcium influx or AA metabolites played a role in controlling respiratory epithelial cell death [34]. Capsaicin, an agonist for TRPV1, can induce airway acute inflammation and moderate respiratory cell dysplasia and necrosis in upper and lower respiratory tracts [35]. In corneal epithelial cells, TPRV1 activation can increase interleukin-6/8 expression and is associated with corneal epithelial cells injury after alkali burn [36]. Since protons have the ability to provoke TRPV1 activation, the exposure to acid in esophageal epithelial cells induces TPRV1 expression and then increases mucosa chemokines secretion, which contributes to esophageal tissue damage [37]. The results of the present study were consistent with this, as the TRPV1 activation in renal tubular cells was associated with a reduction in tubular cell viability. Moreover, IS-induced concentration-dependent increases in ALOX12 and 12(*S*)-HETE expression are responsible for promoting TRPV1 activation, which causes renal tubular damage.

The AhR is a ligand-activated transcription factor that acts as a sensor of xenobiotic chemicals and metabolizes chemical substances, such as polychlorinated biphenyls and dioxins, by upregulating the cytochrome P450 family 1 [38]. Moreover, AhR also acts as a physiological regulator to maintain cellular homeostatic function by modulating cell survival [39]. In the absence of ligand binding, AhR resides within the cytoplasm in a stable complex with heat shock protein 90, heat shock protein 90 cochaperone p23, and X-associated protein 2. Following ligand binding, such as IS, the ligand-receptor complex undergoes transformation and translocates into the nucleus, where it interacts with AhR nuclear translocator protein to form a heterodimer complex. This transformed heteromeric complex is a high-affinity DNA binding form that recognizes a specific DNA sequence, known as a dioxin-responsive element that regulates gene expression by increasing promoter activity [40].

IS is a protein-bound uremic toxin that is normally excreted into the urine. It is produced from the metabolism of dietary L-tryptophan into indole by tryptophanase-expressing gastrointestinal bacteria in the human intestine. After the indole is absorbed into the bloodstream, it is then metabolized into IS by the liver [41]. Prevention of gastrointestinal indole absorption by the IS adsorbent AST-120 to CKD patients is known to lower serum IS levels and delay the initiation of dialysis in patients with advanced-stage CKD [42]. In this study, we also applied AST-120 to the cell culture system and tested whether it can reduce tubular damage in response to IS. However, AST-120 failed to abrogate the effects of IS-induced cell injury, ALOX12, and 12(*S*)-HETE expression (Figure 5). This likely occurred because AST-120 adsorbs indole instead of IS. The systemic activation of AhR signaling by IS is a crucial mediator of uremic toxicity in various tissues and is associated with CKD-related symptoms and complications [43,44,45]. For example, the IS and AhR complex affects cellular immunity dysfunction and is involved in the pathogenesis of atherosclerosis by inducing vascular endothelial cell damage in end-stage renal disease patients [46]. IS also induces inflammatory cytokines in generating oxidative stress that has deleterious cardiovascular effects and exerts nephrotoxicity activity in CKD [47]. In the kidney, AhR expression is distributed in the mature glomerulus, Bowman’s capsules, and renal proximal and distal tubules, but not in the thick segment of the Loop of Henle [48]. Accumulated IS during renal function decline can stimulate glomerular sclerosis and renal tubular dysfunction via AhR and, consequently, CKD progression [8,27,28]. In the AhR-knockout mice, IS fails to enhance leukocyte infiltration into the vascular wall via promoting E-selectin expression [49]. Therefore, AhR is crucial for IS-related cytotoxicity and is compatible with the present findings. After binding to AhR, cytochrome P450 and lipoxygenase activity are subsequently activated, and more lipid derivatives, such as 12(*S*)-HETE, are generated, as demonstrated in this study, further deteriorating inflammation [11]. Eicosanoid synthesis under physiological conditions is important in maintaining normal kidney function. However, upon kidney insults, these derivatives switch their roles in contributing to the pathogenesis of kidney disease [50]. Eicosanoids possess pro- or anti-inflammatory properties and are widely produced by renal tubules that trigger kidney damage in a paracrine fashion [51]. Our results showed that AhR activation by IS increased 12(*S*)-HETE formation via ALOX12, and this contributed to TRPV1 hyperfunction in IS-treated tubular cells.

## 4. Materials and Methods

### 4.1. Tubular Cell Culture and Drug Treatment

As IS is toxic to renal tubules, LLC-PK_1_ and MDCK cells were used as models of proximal and distal tubule cell origin, respectively, as previously described [52]. Cells were obtained from the Bioresource Collection and Research Center (Hsinchu, Taiwan). These cell lines were originally derived from the American Type Culture Collection lines CL-101 (for LLC-PK_1_) and CCL-34 (for MDCK). All culture media and supplements were purchased from Thermo Scientific HyClone (South Logan, UT, USA). LLC-PK_1_ cells were maintained at 37 °C/5% CO_2_ in Medium 199 supplemented with heated inactivated containing 3% fetal bovine serum, sodium bicarbonate (1.5 g/L), penicillin (10,000 U/mL), and streptomycin (10,000 μg/mL). MDCK cells were maintained in Eagle’s Minimum Essential Medium supplemented with heated inactivated containing 10% fetal bovine serum, 2 mM of L-glutamine, 1.5 g/L sodium bicarbonate, 0.1 mM nonessential amino acids, 1 mM sodium pyruvate, and the same concentrations of penicillin and streptomycin. The cells were grown in a humidified incubator at 37 °C in a 5% CO_2_/95% air atmosphere. Cells were maintained and subcultured every 3 days when they reached confluence. For each experiment, the cells were seeded in 1 mL of fresh medium in 24-well plate for 2 days. On the day of the experiment, a 100-μL culture medium of each well was sampled and mixed with chemicals to achieve a final concentration as follows.

Cells were treated with phosphate-buffered saline (PBS, pH 7.4) and IS (2.5, 5.0, 10.0 mM, Merck). Our results showed that MDCK cells were more vulnerable to IS than LLC-PK_1_ cells (Figure 1). The following antagonists, therefore, were treated only in MDCK cells: TRPV1 antagonist capsazepine (Capz, 10 μM, Sigma-Aldrich, St. Louis, MO, USA), a selective TRPV1 antagonist SB-366791 (SB, 5 μM, Tocris, Minneapolis, MN, USA), the uremic toxin adsorbent AST-120 (AST), a specific AhR antagonist CH-223191 (CH, 10 μM, Cayman Chemical, Ann Arbor, MC, USA), selective ALOX12 inhibitors cinnamyl-3,4-dihydroxy-α-cyanocinnamate (CDC, 10 μM, Sigma-Aldrich) and baicalein (Bai, 10 μM, Sigma-Aldrich), or a combination of these drugs for 24, 48, or 72 h. The dosing was determined by reference to the IC_50_ of each antagonist. In another set of experiments, IS was incubated with 2 g/mL AST-120 (Kremezin, Kureha, Tokyo, Japan) for 3 h at 37 °C, as modified from previous reports [53]. The mixture was then centrifuged to remove pellets of AST-120. The supernatants were then filtered and administrated to the cells with an original concentration of IS at 5 mM.

### 4.2. Cytotoxicity Assay

The cytotoxicity effect of IS on LLC-PK_1_ and MDCK cells was determined by a cytotoxicity kit (Roche Applied Science, Mannheim, Germany) that measures lactate dehydrogenase (LDH) release into the culture medium. The absorbance of the mixture solution was measured at 492 nm. The concentration of LDH was quantitated against a standard protein obtained from Sigma-Aldrich, previously described [54,55,56]. The cells’ viability was determined using a 3-(4,5)-2,5-diphenyltetrazolium bromide (MTT) method. Briefly, cells were treated with the indicated concentration of IS alone or in combination with other blockers for 48 h. The culture medium was then removed and washed twice with PBS (pH 7.4). The 5 mg/mL MTT (prepared in PBS) was added to each well and incubated at 37 °C for 4 h. After reaction with MTT, the formazan crystals were then dissolved by dimethyl sulfoxide. The optical density (O.D.) was measured at 570 nm to determine cell viability. The cell viability was calculated as viable cells (%) = (O.D. of treated sample/O.D. of control sample) × 100, as previously described [54,55,56].

### 4.3. Western Blot Analysis for Protein Expression

Cell lysates were prepared as total protein samples using a commercial kit (BioVision, Milpitas, CA, USA), as previously described [16]. Protein amounts in samples were measured using a commercial protein assay kit (Bio-Rad, Hercules, CA, USA) and then separated by sodium dodecyl sulfate-polyacrylamide gel electrophoresis. After electrophoresis, proteins were then transferred to polyvinylidene difluoride membranes, as previously described [16]. The membrane was blocked with 5% skimmed milk and then incubated with the primary antibodies against TRPV1 or ALOX12 (Santa Cruz Biotechnology, Santa Cruz, CA, USA) overnight at 4 °C. After washing, the membranes were then incubated with a corresponding horseradish peroxidase-conjugated IgG (Jackson ImmunoResearch, West Grove, PA, USA) for 1 h at room temperature. The bound antibody complex was visualized using an enhanced chemiluminescence kit (Thermo Scientific, Rockford, IL, USA). The densities of the bands of appropriate molecular masses were determined semi-quantitatively by densitometry using an image analytic system (Diagnostic Instruments, Sterling Heights, MI, USA).

### 4.4. Measurement of 12(S)-HETE Level

The MDCK cells were incubated with the indicated concentration of IS. The cellular content of 12-hydroxyeicosatetraenoic acid (12(*S*)-HETE) was determined using a commercial quantitative immunoassay kit (Biomerica, Newport Beach, CA, USA) according to the manufacturer’s protocol. Homogenized cell extracts, as prepared for Western blotting, were diluted with buffer diluents supplied in the kit, as previously described [16]. The total amount of protein in the sample was quantified using the ELISA immunoassay method. The amount of 12(*S*)-HETE in the cell is expressed as μg per g of protein.

### 4.5. Real-Time Quantitative Polymerase Chain Reaction (RT-qPCR) for the Quantification of TRPV1 and ALOX-12 mRNA Expression

Cellular RNA was isolated using a commercial kit (RareRNA, Bio-East Technology, Taipei, Taiwan), as previously described [56]. The DNase I amplification kit (Invitrogen, Carlsbad, CA, USA) was added to eliminate genomic DNA in RNA solution. Then, cDNA was produced using 5 μg of total RNA, 5 μg of poly(dT)15 oligonucleotide primer (Life Technologies, Carlsbad, CA, USA), and 200 units of reverse transcriptase (Moloney murine leukemia virus; Promega, Madison, WI, USA) at 42 °C for 45 min. A real-time quantitative polymerase chain reaction (RT-qPCR) was performed using an ABI StepOne Plus system (Applied Biosystems, Foster City, CA, USA). PCR was performed in 100 ng of cDNA and 30 μmol of primers in a total reaction volume of 20 μL using the SYBR Green PCR master mix kit, according to the manufacturer’s instructions (Applied Biosystems). The primer used for RT-qPCR lists in Table 1. The PCR was amplified for 20 s at 95 °C and then followed by 40 cycles of 95 °C for 1 s and 60 °C for 20 s. All reactions were run in duplicate. The Δ*C*_t_ (threshold cycle) was calculated by subtracting the raw *C*_t_ values for glyceraldehyde-3-phosphate dehydrogenase (GAPDH) from the raw *C*_t_ values for the target gene, thereby providing information about the relative changes in gene expression. Changes in TRPV1 and ALOX12 expression were calculated as 2^−Δ*C*t^ and expressed as the fold change relative to that of the control group.

## 5. Conclusions

Our results reveal that IS is toxic to renal tubular cells. The cytotoxicity of IS is dependent on the induction of ALOX12 upregulation and its 12(*S*)-HETE production. Inhibition of IS receptor AhR abrogates the increases in ALOX12 and 12(*S*)-HETE, causing more tubular damage after IS treatment. We conclude that TRPV1 hyperfunction caused by the excess of its endogenous ligand 12(*S*)-HETE formation is crucial for IS-induced tubulotoxicity.

## Figures and Tables

**Table 1:** PCR primer sequences in RT-qPCR for the measurement of mRNA expression in cells.

Gene	Sequence
TRPV1	5′-GCG TTT GTC GAC TGA CTG AA-3′ (forward) 5′-CAG GAG TCC AGC TCA CCT TC-3′ (reverse)
ALOX12	5′-AGC TGG AGC CTT TCT GAC CTA TTG-3′ (forward) 5′-ACT GAT TAG GGT TGG GCA GTG TAG-3′ (reverse)
GAPDH	5′-TTA GCA CCC CTG GCC AAG G-3′ (forward) 5′-CTT ACT CCT TGG AGG CCA TG-3′ (reverse)

## Abbreviations

AA	Arachidonic acid
ALOX12	Arachidonate 12-lipoxygenase
12(*S*)-HETE	12-hydroxyeicosatetraenoic acid
12(*S*)-HPETE	12-hydroperoxyeicosatetraenoic acid
ARNA	Afferent renal nerve activity
AhR	Aryl hydrocarbon receptor
Capz	Capsazepine
CDC	Cinnamyl-3,4-dihydroxy-α-cyanocinnamate
CKD	Chronic kidney disease
DRG	Dorsal root ganglia
EMT	Epithelial-to-mesenchymal cell transition
IS	Indoxyl sulfate
LDH	Lactate dehydrogenase
LLC-PK_1_	Porcine proximal tubular cells
MDCK	Madin-Darby canine kidney cells
OAT	Organic anion transporter
PKA	Protein kinase A
PKC	Protein kinase C
ROS	Reactive oxygen species
SB	SB-366791
TRPV1	Transient receptor potential vanilloid 1
